# Low Heart Rate Variability Predicts Poor Overall Survival of Lung Cancer Patients With Brain Metastases

**DOI:** 10.3389/fnins.2022.839874

**Published:** 2022-02-17

**Authors:** Shuang Wu, Guangqiao Li, Weizheng Guan, Huan Zhao, Jingfeng Wang, Yongchun Zhou, Yufu Zhou, Bo Shi

**Affiliations:** ^1^Department of Radiation Oncology, The First Affiliated Hospital of Bengbu Medical College, Bengbu, China; ^2^School of Medical Imaging, Bengbu Medical College, Bengbu, China; ^3^Anhui Key Laboratory of Computational Medicine and Intelligent Health, Bengbu Medical College, Bengbu, China

**Keywords:** heart rate variability, vagus nerve, lung cancer, brain metastases, prognosis

## Abstract

**Background:**

The aim of this prospective study was to evaluate the association between heart rate variability (HRV) and overall survival of lung cancer patients with brain metastases (LCBM).

**Methods:**

Fifty-six LCBM patients were enrolled in this study. Five-minute electrocardiograms were collected before the time to first brain radiotherapy. HRV was analyzed quantitatively by using the time domain parameters, i.e., the standard deviation of all normal-normal intervals (SDNN) and the root mean square of successive differences (RMSSD). Survival time for LCBM patients was defined as from the date of HRV testing to the date of death or the last follow-up.

**Results:**

In the univariate analysis, SDNN ≤ 13 ms (*P* = 0.003) and RMSSD ≤ 4.8 ms (*P* = 0.014) significantly predicted poor survival. Multivariate analysis confirmed that RMSSD ≤ 4.8 ms (*P* = 0.013, hazard ratio = 3.457, 95% confidence interval = 1.303–9.171) was also an independent negative prognostic factor after adjusting for mean heart rate, Karnofsky performance status, and number of brain metastases in LCBM patients.

**Conclusion:**

Decreased RMSSD is independently associated with shorter survival time in LCBM patients. HRV might be a novel predictive biomarker for LCBM prognosis.

## Introduction

According to 2020 global cancer statistics, lung cancer (LC) is the second most common malignancy, and its mortality rate ranks first in the world (Sung et al., 2021). One of the common and serious complications of LC is brain metastases (BM) (Moravan et al., 2020). It is conservatively estimated that approximately 10 ∼ 30% of LC patients will develop BM (Sperduto et al., 2017). The common treatment methods for LC patients with BM (LCBM) include surgical resection, whole brain radiotherapy, stereotactic radiosurgery, etc. (Kotecha et al., 2018). Advances in treatment technology have prolonged the survival time of LCBM to some extent, but the overall prognosis of patients is still poor, with a 2-year overall survival (OS) rate of only 8.1% (Hall et al., 2000). Decisions regarding precision therapy for LCBM are commonly faced by clinicians, and effective prognostic assessment is a prerequisite for guiding precision treatment (Sperduto et al., 2008; Suh et al., 2020).

Numerous prognostic models, i.e., recursive partitioning analysis (RPA) (Gaspar et al., 1997), graded prognostic assessment (GPA) (Sperduto et al., 2008), and graded prognostic assessment for lung cancer with brain metastases using molecular markers (lung-molGPA) (Nieder et al., 2017), have been developed for prognostic evaluation and survival analysis of LCBM patients. The above models are mainly composed of important prognostic factors such as age, Karnofsky performance status (KPS), number of BM, information about the primary tumor, and the presence and status of extracranial disease (Gaspar et al., 1997; Sperduto et al., 2008; Nieder et al., 2017). However, the determination of these factors is challenging due to low certainty and high subjectivity, which weaken the predictive effect of the model. Therefore, it is necessary to develop a more effective, objective and convenient prognostic assessment method.

Studies have shown that the autonomic nervous system (ANS) is related to the occurrence and development of tumors (Faulkner et al., 2019). Excessive sympathetic nervous system activity is found to promote cancer growth and the development of metastasis (Park et al., 2011; Zhi et al., 2019); however, vagus nerve (VN) stimulation can inhibit excessive sympathetic activity (Saku et al., 2014) inflammation (Tracey, 2009) and increase cellular immunity (Dubeykovskaya et al., 2016). Therefore, high VN activity may predict longer OS and have a protective role in cancer patients (Gidron et al., 2005; De Couck et al., 2012).

Heart rate variability (HRV) is a non-invasive biomarker for quantitative evaluation of ANS activity (Camm et al., 1996; Lombardi and Stein, 2011). In the study of HRV and survival of BM patients, Wang et al. (2013) indicated that BM patients with the standard deviation of all normal-to-normal intervals (SDNN) < 10 ms had a significantly lower OS than those with SDNN ≥ 10 ms. In addition, Wang et al. (2021) reported in another study that SDNN < 10 ms was an independent prognostic factor for OS in non-elderly BM patients (*P* = 0.010; hazard ratio = 2.664). The results of the studies performed by Wang et al. (2013, 2021) indicated that time-domain HRV may be identified as a prognostic marker for BM patients, but their two studies did not consider the confounding factors affecting HRV, including body mass index (BMI), mean heart rate (Mean HR), respiration rate (RR), etc. Moreover, the participants included in these studies had primary malignant tumors such as LC and breast cancer (BC). There was obvious heterogeneity among these cancers, which may weaken the evaluation of the effect of HRV. Therefore, this study took LCBM as the research object and tested whether time-domain HRV indices were independently associated with survival time in LCBM patients after adjusting for confounding factors.

## Materials and Methods

### Subjects

In this prospective study, patients diagnosed with LCBM who received radiotherapy for the first time at the First Affiliated Hospital of Bengbu Medical College (Bengbu, Anhui, China) were recruited between October 2019 and April 2021. All participants were diagnosed with LC by pathological examination and diagnosed with BM using enhanced computed tomography or magnetic resonance imaging. The following exclusion criteria were applied: (1) presence of a pacemaker; (2) use of anti-arrhythmic drugs or beta-blockers; (3) leptomeningeal metastasis; or (4) previous brain radiotherapy or surgery. This study followed the STROBE checklist for reporting required information (see checklist^1^). This study obtained the approval of the Institutional Review Board (IRB) of the First Affiliated Hospital of Bengbu Medical College (IRB number: 2019KY031). This research strictly adhered to the ethical standards set forth in the Declaration of Helsinki and its amendments in June 1964. All patients were informed of the detailed purpose, procedure, risks and adverse reactions of the experiment and signed consent before enrolment.

### Data Collection

The resting electrocardiogram (ECG) data of subjects in the supine position were collected in a quiet and undisturbed room at a room temperature of 23 ± 1°C, and signal collection lasted 5 min. An ECG recorder (HeaLink-R211B; HeaLink Ltd., Bengbu, China) was used to collect ECG signals at a 400 Hz sampling rate and V6-lead. Before signal recording commenced, the participants were informed of the data collection procedure and asked to remain still, to not talk, and to breathe regularly and gently during ECG collection.

### Heart Rate Variability Analysis

Based on the Pan-Tompkins algorithm, the ECG R-R interval time series was extracted (Pan and Tompkins, 1985). An ECG-derived respiration rate was calculated by the ECG signal (Moody et al., 1985).

Time- and frequency-domain analyses are frequently used for HRV analysis (Camm et al., 1996), and time-domain and frequency-domain indices are relevant. However, compared with the time-domain analysis method, the frequency-domain analysis method of HRV has some problems. First, the power spectral density (PSD) analysis results can be greatly affected by the spikes caused by the artifacts and ectopic pacing points in HRV (Thireau et al., 2008). Second, PSD analysis mainly includes fast Fourier transform and autoregressive model methods. There is no fixed standard for the method and parameter setting, limiting its practical application (Laguna et al., 1998; Clifford and Tarassenko, 2005). Therefore, this study used the time-domain analysis method of HRV, which is simple to calculate. SDNN and the root mean square of successive differences (RMSSD) were used for HRV time-domain analysis to assess the prognosis of LCBM (Camm et al., 1996; Lombardi and Stein, 2011). SDNN well describes overall HRV, reflecting both sympathetic and parasympathetic activity, but it is impossible to distinguish whether the decreased SDNN is due to diminished vagal or increased sympathetic modulation (Sztajzel, 2004; Vanderlei et al., 2009). A precise understanding and consensus on the autonomic underpinnings of SDNN has not yet been achieved. Compared with SDNN, the physiological significance of RMSSD is clearer. RMSSD is considered to be the most suitable method to quantify short-term HRV, which represents parasympathetic activity in short-term records independent of day/night variations (Sztajzel, 2004; Vanderlei et al., 2009). The above HRV indices were analyzed by Kubios HRV Premium software (version 3.1.0^2^, Kubios Oy, Kuopio, Finland) (Niskanen et al., 2004).

### Follow-Up

The survival time of LCBM patients was defined as the date of HRV collection to the date of death or the date of the last follow-up. The follow-up method was telephone follow-up, and the last follow-up date was September 25, 2021. There was no loss to follow-up.

### Statistical Analysis

The estimation of the sample size was based on the two previously published studies (Wang et al., 2013, 2021) about the association between HRV and survival in BM patients and no specific statistical method was used. We added 40% (*n* = 56) based on the Wang et al. (2013, 2021) sample size (*n* = 40). Normal or non-normal continuous data are expressed as the mean (standard deviation) and median (1st quartile, 3rd quartile), respectively, while categorical data are expressed as frequencies and percentages. For the differences of parameters between survival and non-survival groups, the unpaired Student’s *t*-test and Mann–Whitney *U* test were used to analyze the continuous data and the counting data were analyzed by Chi-square test. The optimal cut-off values that could evaluate the survival time of patients were obtained by X-tile software (Robert L. Camp, Yale University, New Haven, Connecticut, United States), and the corresponding HRV indices were classified into two categories. X title software allows for a single cohort to be divided into training and validation subsets for *P* value estimation when separate training and validation cohorts are not available and performs standard Monte Carlo simulations (for example, cross-validation) to generate corrected *P* values to assess the statistical significance of data evaluated by multiple cut-off points (Camp et al., 2004). The Kaplan–Meier method was used to estimate median survival time and construct time-to-event curves. The following clinical factors were included in this study according to previously published articles: sex, age, BMI, KPS, pathological type, primary tumor status, the presence of extracranial metastases, number of BM, and whether systemic treatment was performed after BM (Gaspar et al., 1997; Sperduto et al., 2008; Nieder et al., 2017). The clinical factors and factors affecting HRV, including Mean HR and RR, were included in the univariate Cox regression analysis to identify statistically significant prognostic confounding factors. Due to the correlation among HRV indicators, to evaluate the independent prognostic HRV indices affecting LCBM patients, we performed a multivariate Cox regression analysis for each HRV parameter individually with the prognostic confounding factors that were shown to be significant in the univariate analysis. There were no missing data in this analysis for all variables. The above statistical analysis was performed by SPSS Statistics 25.0 (IBM Corp., Chicago, Illinois, United States of America), and statistical significance was defined as a 2-tailed *P* value < 0.05.

## Results

Overall, 56 patients diagnosed with LCBM were included in this study. The general characteristics of the participants and comparison of parameters between non-survived and survived groups are shown in Table 1. The study included 19 females and 37 males. The average age of patients at ECG collection was 60.4 ± 9.0 years. More than half of the patients had KPS > 70 (*n* = 39). Extracranial metastases were found in 27 patients. Forty-seven patients presented with two or more brain metastases. The pathological types of LC were NSCLC (*n* = 40) and SCLC (*n* = 16). The status of primary tumors was uncontrolled (*n* = 42) and controlled (*n* = 14). System therapy was performed in 32 patients even after the diagnosis of BM. Thirty-two (57.1%) patients died, and 24 (42.9%) patients were still alive with a median follow-up of 9.3 months (range 1.0–23.3 months).

**TABLE 1:** General characteristics of all patients and comparison of parameters between non-survived and survived groups.

	All (*N* = 56)	Non-survivors (*N* = 32)	Survivors (*N* = 24)	*P* value
Sex				0.290
Female/Male	19/37	9/23	10/14	
Age (year)	60.4 ± 9.0	62.0 [53.3, 66.8]	63.5 [51.3, 67.0]	0.810
BMI (kg/m^2^)	22.8 ± 3.3	22.0 ± 3.4	23.9 ± 3.0	**0.039**
Mean HR (bpm)	79.41 ± 12.91	81.75 ± 13.43	76.29 ± 11.73	0.118
RR (Hz)	0.31 ± 0.07	0.32 ± 0.07	0.30 ± 0.07	0.187
KPS				**0.002**
≤ 70/ > 70	17/39	15/17	2/22	
Pathological type				0.932
NSCLC/SCLC	40/16	23/9	17/7	
Extracranial metastasis				0.757
Without/With	29/27	16/16	13/11	
Primary tumor status				1.000
Not controlled/Controlled	42/14	24/8	18/6	
Number of BM				0.052
Single/Multiple	9/47	2/30	7/17	
Systemic treatment after BM				0.876
Without/With	24/32	14/18	10/14	
SDNN (ms)	19.15 ± 9.94	18.24 ± 10.82	20.36 ± 8.7	0.434
RMSSD (ms)	10.15 [6.25, 15.20]	8.85 [6.00, 12.88]	10.70 [7.08, 17.33]	0.285

*BM, brain metastasis; BMI, body mass index; Mean HR, mean heart rate; bpm, beats per minute; RR, respiration rate; KPS, Karnofsky performance status; NSCLC, non-small cell lung cancer; SCLC, small cell lung cancer; SDNN, standard deviation of all normal-to-normal intervals; RMSSD, root mean square of successive differences; SD, standard deviation. Values are expressed as the number of patients, mean ± SD or median [Q1, Q3]. Bold P values indicate statistical significance (P value < 0.05).*

We conducted a univariate analysis to identify the basic clinical prognostic factors of survival in LCBM patients and found that Mean HR, KPS, and the number of BM were significantly associated with survival in patients with LCBM [Mean HR: hazard ratio = 1.044, 95% confidence interval (CI): 1.013–1.076, *P* = 0.005; KPS: hazard ratio = 2.769, 95% CI 1.373–5.584, *P* = 0.004; number of BM: hazard ratio = 0.229, 95% CI: 0.054–0.967, *P* = 0.045]. The associations of survival time with sex, age, BMI, RR, pathological type of LC, primary tumor status, extracranial metastases, and systemic treatment after BM were not significant in the univariate analysis (Table 2).

**TABLE 2:** Univariate analysis of the baseline patient characteristics associated with survival.

Variables	Univariate analysis	
	Hazard ratio (95% CI)	*P* value
**Sex**		
Female	0.763 (0.350, 1.667)	0.498
Male	Ref	
Age (year)	0.999 (0.960, 1.039)	0.946
BMI (kg/m^2^)	0.911 (0.815, 1.108)	0.099
Mean HR (bpm)	1.044 (1.013, 1.076)	**0.005**
RR (Hz)	124.235 (0.963, 16,029.829)	0.052
**KPS**		
≤70	2.769 (1.373, 5.584)	**0.004**
>70	Ref	
**Pathological type**		
NSCLC	1.202 (0.541, 2.667)	0.652
SCLC	Ref	
**Extracranial metastasis**		
Without	1.016 (0.506, 2.039)	0.965
With	Ref	
**Primary tumor status**		
Not controlled	0.924 (0.414, 2.063)	0.848
Controlled	Ref	
**Number of BM**		
Single	0.229 (0.054, 0.967)	**0.045**
Multiple	Ref	
**Systemic treatment after BM**		
Without	1.171 (0.574, 2.386)	0.664
With	Ref	

*BM, brain metastasis; BMI, body mass index; bpm, beats per minute; CI, confidence interval; Mean HR, mean heart rate; RR, respiration rate; KPS, Karnofsky performance status; NSCLC, non-small cell lung cancer; SCLC, small cell lung cancer. Bold P values indicate statistical significance (P value < 0.05).*

**FIGURE 1:**
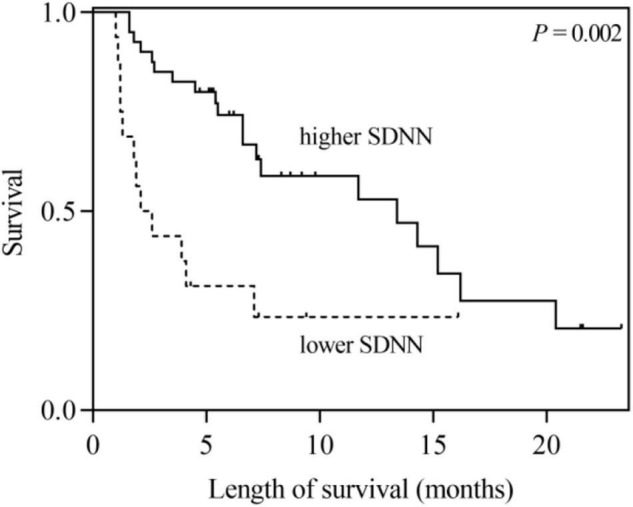
Kaplan–Meier survival curves for LCBM patients with SDNN ≤ 13 ms versus those with SDNN > 13 ms.

**FIGURE 2:**
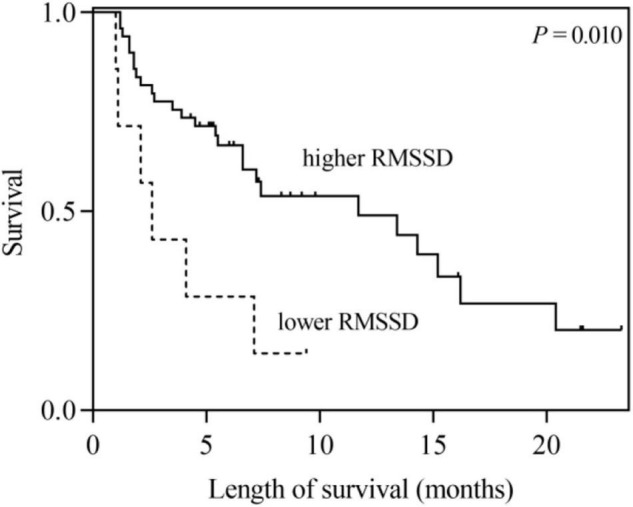
Kaplan–Meier survival curves for LCBM patients with RMSSD ≤ 4.8 ms versus those with RMSSD > 4.8 ms.

According to the univariate analysis, the time-domain indices of HRV were shown to be significantly associated with survival time in LCBM. The median survival in LCBM patients with SDNN > 13.0 ms and SDNN ≤ 13.0 ms was 13.4 and 2.1 months (*P* = 0.003), respectively. Patients with RMSSD > 4.8 ms had a longer survival time than those with RMSSD ≤ 4.8 ms (11.7 vs. 2.6 months, *P* = 0.014) (Table 3 and Figures 1, 2). After adjusting for confounders in multivariate analysis, the RMSSD of time-domain HRV indices was an important independent prognostic factor for the survival of LCBM patients (hazard ratio = 3.457, 95% CI: 1.303–9.171, *P* = 0.013). However, there was no significant association between SDNN and survival time (Table 3).

**TABLE 3:** Univariate and multivariate analysis of heart rate variability variables as predictors of survival.

Variables	Median survival (M)	Univariate analysis		Multivariate analysis	
		Hazard ratio (95% CI)	*P* value	Hazard ratio (95% CI)	*P* value
**SDNN (ms)**					
≤13.0	2.1	3.100 (1.477, 6.504)	**0.003**	2.408 (0.957, 6.058)	0.062
>13.0	13.4	Ref		Ref	
**RMSSD (ms)**					
≤4.8	2.6	3.151 (1.259, 7.884)	**0.014**	3.457 (1.303, 9.171)	**0.013**
>4.8	11.7	Ref			

*CI, confidence interval; SDNN, standard deviation of all normal-to-normal intervals; RMSSD, root mean square of successive differences. Bold P values indicate statistical significance (P value < 0.05).*

## Discussion

In this research, we explored the association between time-domain HRV parameters and the survival of LCBM patients. The results showed that SDNN and RMSSD were significantly correlated with the survival duration in LCBM patients in the univariate analysis. After adjusting for confounders, including Mean HR, KPS and the number of BM, RMSSD was found to be an independent prognostic factor for OS in patients with LCBM in the multivariate analysis.

Previous studies have shown that SDNN significantly correlates with the survival of patients with malignancy. De Couck et al. (2016) and Guo et al. (2015) found that a higher SDNN could predict a longer survival duration among patients with malignancy. For patients with BM, Wang et al. (2013, 2021) showed that SDNN was an independent prognostic factor after correcting confounding factors such as KPS. In this study, we found that SDNN was significantly related to the survival time of LCBM patients in the univariate analysis (hazard ratio = 3.100, 95% CI: 1.477–6.504, *P* = 0.003). However, after adjusting for Mean HR, KPS and the number of BM, the association between SDNN and survival time tended to be significant (hazard ratio = 2.408, 95% CI: 0.957–6.058, *P* = 0.062). This may be related to our consideration of the confounding factors of Mean HR.

Numerous clinical prognosticators have been used to evaluate the survival of cancer patients. Previous researchers have identified age, KPS, information about extracranial disease and the number of BM as important prognostic factors (Gaspar et al., 1997; Sperduto et al., 2008; Nieder et al., 2017). Our univariate analysis results showed that the KPS and number of BM significantly affected the survival duration of LCBM patients (KPS: hazard ratio = 2.769, 95% CI: 1.373–5.584, *P* = 0.004; number of BM: hazard ratio = 0.229, 95% CI: 0.054–0.967, *P* = 0.045), further confirming the validity of traditional clinical prognostic factors. However, confounding factors (such as Mean HR and RR) significantly related to HRV were not taken into account in the above studies (Wang et al., 2013, 2021; Guo et al., 2015; De Couck et al., 2016). It has been confirmed that HRV is significantly correlated with Mean HR in healthy people and people with some diseases (Sacha, 2013; Monfredi et al., 2014). Mean HR and RR were included as confounding factors in our study, and the univariate analysis showed that Mean HR had a significant correlation with survival time (hazard ratio = 1.044, 95% CI: 1.013–1.076, *P* = 0.005). In the multivariate analysis including Mean HR, SDNN was no longer significantly associated with the survival of patients with LCBM.

RMSSD regulated by the parasympathetic nervous system is an important marker of VN activity (Sztajzel, 2004; Vanderlei et al., 2009). Recent observational studies have shown that RMSSD may provide useful prognostic information in clinical settings. De Couck et al. (2013) confirmed that RMSSD significantly predicted the survival time of patients with non-small cell lung cancer (NSCLC) in individuals younger than 65 years of age. Bijoor et al. (2016) showed that compared to healthy individuals, the RMSSD was significantly lower in cancer patients, and the RMSSD of the advanced cancer group was significantly lower than that of the early cancer group. Arab et al. (2018) found that the RMSSD of the advanced BC group was lower than that of the early BC group and benign breast tumor group, while there was no significant difference in RMSSD between the early BC group and benign breast tumor group. The results of the above studies demonstrated that higher VN activity (characterized by RMSSD) is associated with disease progression and survival in cancer patients.

In addition, the high-frequency power (HF) of the HRV frequency-domain index is highly correlated with RMSSD, which also reflects VN activity (Sztajzel, 2004; Vanderlei et al., 2009). Chiang et al. (2010) first evaluated the association between ANS function and time-to-death (TTD) in patients with terminal hepatocellular carcinoma. The results showed that there was a significant correlation between HF and TTD (*r* = 0.44, *P* = 0.010). Subsequently, a prospective study of 138 patients with advanced cancer conducted by Chiang et al. (2013) revealed that HF was closely related to the survival of 7 days or less for hospice patients with non-lung cancer. Moreover, Giese-Davis et al. (2015) also obtained a similar conclusion: among patients with metastatic or recurrent BC, the survival time of patients with higher baseline HF was significantly longer than that of patients with lower HF. The results of the above studies have shown that especially in advanced cancer patients, VN is closely related to the OS of cancer patients. The results of our study indicated that low RMSSD was an independent risk factor for poor prognosis in LCBM patients (hazard ratio = 3.457, *P* = 0.013), further confirming that high VN activity predicted longer survival in LCBM patients. Wang et al. (2013) did not find an association between RMSSD and the survival of BM patients, which may be due to the large number of primary tumor types (LC, BC, etc.) included in their study. Because of the obvious heterogeneity among these tumors, the evaluation of the effect of HRV was undermined.

Accurate prognosis prediction of cancer patients can help guide clinical decision making. Our study showed that RMSSD, a time-domain HRV parameter that characterizes VN activity, is an independent prognostic factor for LCBM patients. A systematic review conducted by Palma et al. (2020) showed the impact of supportive therapy on the VN activity (assessed by HRV analysis) of cancer patients. The results indicated that supportive therapy (including music therapy intervention, traditional Chinese medicine-related therapy and exercise intervention) may enhance ANS function. If VN stimulation proves to be effective in future prospective randomized controlled trials, then improvements in VN tone may benefit patients with advanced cancer and may become a potential treatment approach in the future. VN stimulation therapy, such as electroacupuncture from traditional Chinese medicine, is likely to be used as part of comprehensive cancer treatment strategies in the future (Liu et al., 2020, 2021).

### Limitations

Although this is a prospective study, several limitations still exist. First, twenty-four (42.9%) were alive at the end of this study, and our results still need to be further validated by more long-term follow-up studies. Second, there was heterogeneity in the radiotherapy modality and dose received by patients, and due to the small sample sizes of the different subgroups, it was impossible to statistically analyze the impact of radiotherapy methods and doses on the survival of LCBM patients. Finally, because the number of BM in some patients was unclear, we could not use GPA as a prognostic index in the survival analysis of LCBM patients. Future independently prospective studies with further expansion of samples size are required, especially further investigation of the correlation between vagal activity/vagal stimulation and survival time in patients with LCBM.

## Conclusion

This study revealed that RMSSD is independently related to the survival time in patients with LCBM, which may indicate that the VN plays an important role in the prognosis of LCBM patients. The results of the current study should be verified by an independently prospective study with larger sample sizes and long-term follow-up. If these findings can be verified, it may be possible to tailor personalized treatment strategies and perform prognostic evaluations based on patient vagal activity.

## Data Availability Statement

The original contributions presented in the study are included in the article/supplementary material, further inquiries can be directed to the corresponding authors.

## Ethics Statement

The studies involving human participants were reviewed and approved by The First Affiliated Hospital of Bengbu Medical College. The patients/participants provided their written informed consent to participate in this study.

## Author Contributions

BS: conceptualization, methodology, resources, and writing—review and editing. YFZ: supervision and resources. SW: data collection and writing—original draft preparation. GL: data analysis and writing—original draft preparation. WG, HZ, and JW: writing—original draft preparation. YCZ: supervision. All authors contributed to the article and approved the submitted version.

## Conflict of Interest

A direct family member of BS owns stock in HeaLink Ltd., Bengbu, China. The remaining authors declare that the research was conducted in the absence of any commercial or financial relationships that could be construed as a potential conflict of interest.

## Publisher’s Note

All claims expressed in this article are solely those of the authors and do not necessarily represent those of their affiliated organizations, or those of the publisher, the editors and the reviewers. Any product that may be evaluated in this article, or claim that may be made by its manufacturer, is not guaranteed or endorsed by the publisher.

## Footnotes

1
https://www.strobe-statement.org/

2
https://www.kubios.com
